# Palaeomagnetism of the Upper Miocene- Lower Pliocene lavas from the East Carpathians: contribution to the paleosecular variation of geomagnetic field

**DOI:** 10.1038/srep23411

**Published:** 2016-03-21

**Authors:** Mădălina Vişan, Cristian G. Panaiotu, Cristian Necula, Anca Dumitru

**Affiliations:** 1 Institute of Geodynamics, Romanian Academy, Jean-Luis Calderon 19-21, 020032, Bucharest, Romania; 2 Paleomagnetic Laboratory, Faculty of Physics, University of Bucharest, Atomiştilor 405, Măgurele, Ilfov, Romania

## Abstract

Investigations of the paleosecular variation of the geomagnetic field on geological timescales depend on globally distributed data sets from lava flows. We report new paleomagnetic results from lava flows of the East Carpathian Mountains (23.6°E, 46.4°N) erupted between 4 and 6 Ma. The average virtual geomagnetic pole position (76 sites) includes the North Geographic Pole and the dispersion of virtual geomagnetic poles is in general agreement with the data of the Time Averaged geomagnetic Field Initiative. Based on this study and previous results from the East Carpathians obtained from 0.04–4 Ma old lava flows, we show that high value of dispersion are characteristic only for 1.5–2.8 Ma old lava flows. High values of dispersion during the Matuyama chron are also reported around 50°N, in the global paleosecular variation data set. More data are needed at a global level to determine if these high dispersions reflect the behaviour of the geomagnetic field or an artefact of inadequate number of sites. This study of the East Carpathians volcanic rocks brings new data from southeastern Europe and which can contribute to the databases for time averaged field and paleosecular variation from lavas in the last 6 Ma.

The Earth’s magnetic field is generated by geodynamo processes in the Earth’s liquid outer core and its direction and strength vary with time^1^. The range of field variability relevant to this paper occurs on timescales of years to millennia, during stable polarity epochs, and is known as geomagnetic secular variation. Studies dedicated to paleosecular variation (PSV) of the earth’s magnetic field during the last 5 Ma have been considerably developed in the last 10–15 yr^2^,^3^,^4^,^5^,^6^. Most of these studies have introduced more rigorous criteria^2^ to select the data than those used in previous PSVstudies^1^. Lava flows are the best source of data, because volcanics provide geologically instantaneous recordings of field behaviour, without the temporal averaging inherent in sedimentary records^2^. Investigations of the behaviour of the geomagnetic field on geological timescales are based on globally distributed data sets from dated lava flows. The compilations of global data sets often suffer from limited geographical coverage, inadequate or poorly documented temporal sampling, or poor data quality^2^. In particular, an improved database could allow the detection of any temporal dependency of PSV, as observed for the Brunhes and Matuyama chrons^2^.

In the eastern Carpathian–Pannonian region during the last 15 Ma, westward-dipping subduction in a land-locked basin caused collision of a lithospheric block from the west with the southeastern border of the European plate^7^,^8^. After the main collisional events at 11 Ma^9^, volcanism took place in the East Carpathians forming the Călimani – Gurghiu – Harghita (CGH) volcanic chain (Fig. 1). This volcanic chain consists of calc-alkaline products that occurred along the easternmost margin of the rigid Transylvanian block, in the front of European Platform and it marks the end of the post-collisional subduction-related magmatism along the front of the European convergent plate margin^10^. Its generation may be associated with asthenosphere uprise, explained by progressive break-off of the Miocene subducted slab^10^. The volcanic chain consists of a NW–SE-trending row of closely spaced adjacent volcanic edifices (Fig. 1). Most of them are medium-size composite volcanoes and a couple of them with summit calderas^11^. The CGH volcanic chain is around 160 km long and the volcanic activity gradually migrated to the south between the Upper Miocene (~10 Ma) and the Quaternary (~0.03 Ma)^12^. Due to this migration the CGH post-collisional volcanic chain is an excellent place to study the PSV from lava flows over the last 10 Ma in time steps of 1–2 Ma.

Previous paleomagnetic results from the CGH volcanic chains were obtained from lava flows younger than 4 Ma^13^,^14^. In this paper we present new paleomagnetic results obtained from volcanic rocks erupted between 4 and 6 Ma. This area was also studied in earlies 70s, but this pioneering paleomagnetic study^15^ for the East Carpathians does not fulfil any more the modern paleomagnetic quality standards for PSV studies. For this reason we sampled 84 new sites in the main volcanic structures from the North Harghita Mountains (Vârghiş, Ivo-Cocoizaş and Ostoroş) and a small volcanic structure from the southern tip of the Gurghiu Mountains (Supplementary Fig. S1). The northern volcanic structures (Ostoroş and Ivo-Cocoiaş) are eroded volcanoes with intrusive core complexes and rim domes, consisting of basaltic andesitic, andesitic lavas and very minor pyroclastic^11^. The rest of North Harghita is occupied by the large and complex Vârgiş volcano. This volcano had initially an intense volcano building stage, followed by a large edifice failure/debris avalanche event and second-stage effusive activity^11^. Andesitic and andesitic dacitic lavas and andesitic pyroclastic rocks are the main volcanic products from the Vârgis volcanic structure^11^. Twenty five K-Ar ages^16^ have shown that the volcanism has started around 6 Ma in the northern volcanic structures (Ivo-Cocoiaş, Ostoroş and Răchiţis) and migrated toward south after 1 Ma forming the Vârghiş volcanic structures, where the volcanism was active until ~4 Ma (Supplementary Fig. S2). The North Harghita Mountains are covered with forest, so the number of outcrops suitable for a palaeomagnetic study is limited and lava flow successions are not available for sampling. The samples were collected only from outcrops in andesitic, basaltic andesitic and andesitic dacitic lava flows. The suitable outcrops were chosen to be chemically unaltered as possible and *in situ* according to field observations. In order to avoid the sampling of the same lava for multiple times, the sites were separated by hundreds of metres or more and/or at different elevations. Sampling was carried out by drilling cores 2.5 cm in diameter using a handheld gasoline-powered engine with a water pump. The samples were oriented using a Brunton magnetic compass and a sun compass where possible. Each site consisted of 6–10 samples.

## Results

### Rock magnetism

**Figure 2:**
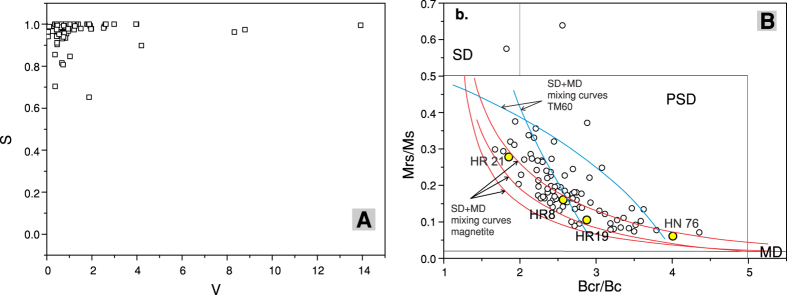
(**A**) S ratio vs. V parameter; (**B**) Day plot of site-representative samples. The boundaries between SD, PSD and MD regions and SD–MD mixing curves for magnetite (red lines) and TM60 (blue lines) are after Dunlop^20^. Yellow dots mark the position of samples with FORC diagrams presented in Supplementary Fig. S4.

Most of the sites (91 per cent) have *S* ratio above 0.90, indicating a ferromagnetic mineralogy dominated by low coercivity magnetic minerals. For most of the samples, field dependence of magnetic susceptibility (V parameter) shows no or a very small variation (Fig. 2A). This behaviour is compatible with the presence of magnetite and/or titanium-poor titanomagnetite^17^. Low-field susceptibility versus temperature experiments were conducted to determine the Curie temperature (T_C_) of selected specimens (Supplementary Fig. S3). All samples exhibit a T_C_ between 550 and 580 °C, which indicates the presence of magnetite. In some samples we observed a decay of magnetic susceptibility between 350 and 450 °C. This drop was attributed to the inversion of maghemite^18^.

The Day-plot^19^ (Fig. 2B) shows that the majority of samples plot in the pseudo-single domain (PSD) range, between the single domain (SD) – multidomain (MD) magnetite and TM60 theoretical mixing curves^20^. The First Order Reversal Curves (FORC) diagrams (Supplementary Fig. S4) are compatible with a mixture of SD and MD grains^21^,^22^, in agreement with theoretical models, which show that PSD behaviour is due to superimposed independent SD and MD moments^20^. The closed contours with a well-defined central peak suggest the presence of non-interacting or weakly interacting SD particles and the contours diverging away from the origin indicate the presence of MD particles^21^,^22^. There is a gradual increase of the MD grains contribution as we move along the SD-MD magnetite mixing curves (Fig. 2b and Supplementary Fig. S4).

## Directional Results

Natural remanent magnetization and demagnetization behaviour were measured on a total of 535 independent samples from 84 sites. Typical step-wise alternating fields (AF) and thermal demagnetization patterns are presented in Supplementary Fig. S5. The results of thermal demagnetization are indistinguishable from those obtained from other specimens at the same site using AF demagnetization. The distribution of blocking temperatures is in agreement with the ferromagnetic mineralogy revealed by rock magnetic measurements. AF demagnetization was the preferred technique for magnetically cleaning the rest of the collection. In most samples, demagnetization data show that fields of 10–20 mT or temperature below 250 °C were sufficient to remove a weak viscous component and successive higher fields produced linear principal component vectors that trend toward the origin. The line fits were based on the following constrains: (1) minimum four demagnetization steps; (2) the line fit was anchored to the origin using principal component analysis^23^ and the Remasoft 3.0 software^24^; (3) the maximum angular deviation was less than 10° (usually less than 5°). Three sites sampled around the Mădăraş Peak (1801 m altitude, the highest peak in the North Harghita Mountains) have very high values of the natural remanent magnetization (2–40 A/m) and are very soft with the median destructive field of 10–20 mT. We presume that these sites are affected by lighting^25^. One of the sites from the same area differs from the others, because the demagnetization data shows a complicated multicomponent behaviour, probably given by several successive lightning strikes near the site. All these four sites were not considered for the data analysis. Other two sites from the southern part of the Vârghiş volcanic structure showed an unstable and chaotic demagnetization path and they have not been also used in this study.

**Figure 3:**
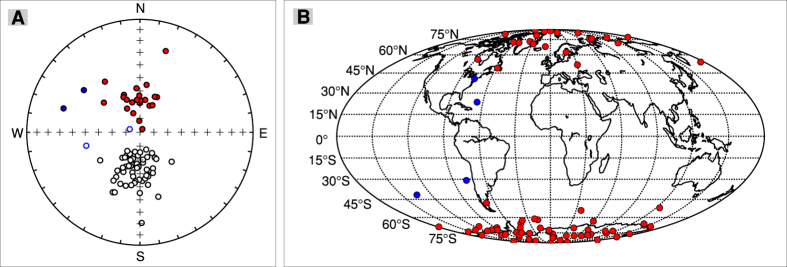
(**A**) Equal area projection of site mean directions of lava flows erupted between 4 and 6 Ma. The full circles and open circles represent the lower and upper hemisphere directions, respectively. Blue circles represent the sites removed by the latitude cut-off of 45°; (**B**) Mollweide projection of VGPs. Blue circles represent the sites removed by the latitude cut-off of 45°. Both projections were created using the PmagPy-3.24 software package^32^ (http://earthref.org/PmagPy/cookbook/).

The mean site directions were calculated using Fisher statistics^26^ by averaging the AF and thermal results. A summary of site mean directions and virtual geomagnetic poles (VGPs) are presented in Supplementary Table S1 and plotted in Fig. 3. The areal distribution of sites with normal and reversed polarities (Supplementary Fig. S1) is in agreement with geographic and time distribution of K-Ar ages (Supplementary Fig. S2) and polarity timescale^27^. The results showed that the northern volcanic structures (Ivo-Cocoiaş and Ostoroş) have only sites with reversed polarity. These sites were probably emplaced mainly during chron C3r (6.0–5.2 Ma), which have only reversed polarity. The results from the Vârghiş volcanic structure showed the presence of sites with reversed and normal polarity. Taking into account the K-Ar ages, the volcanism took place mostly during chron C3n (5.2–4.1 Ma), which has mixt polarities. The geographic distribution of the magnetic polarities supports the available K-Ar ages and show that the volcanism started in the northern part of the area and migrated toward the south^12^.

## Discussions

To analyse the PSV between 4 and 6 Ma we have used the new data from the Northern Harghita Mountains and 7 sites from the norther part of the South Harghita Mountains^13^, which have an age greater than 4 Ma^16^ (Supplementary Table S1 and Supplementary Fig. S1). Only the sites which fulfil the quality selection criteria for PSV studies^2^ have been used in this analysis: n (number of samples) ≥ 5 and k (precision parameter from Fisher statistics) ≥ 50. Taking into account these criteria we have selected 80 sites from 85 (cf. Supplementary Table S1). Most sites (79) also α_95_ ≤ 10°, which is in agreement with cut-off value of α_95_ proposed for PSV studies^4^. No attempt was made to filter the data of serial correlation in lavas, because similar directions were not required due exclusively to rapid sequential depositions of lava flows, but they can be also produced by slow secular variation^28^.

The next step in the analysis of PSV was the identification of sites which can be categorized as transitional directions associated with low VGPs latitudes. Two methods were used to identify these sites: (1) a constant VGP latitude cut-off of 45° 2; (2) the variable latitude cut-off based on the Vandamme criterion^29^. The first method, the constant VGP latitude cut-off of 45°, has removed four sites (Fig. 3). Using the Vandamme criterion resulted in a latitude cut-off value of 56°, which removed another three sites. We decided to use the results obtained using the latitude cut-off of 45° because it is less restrictive and facilitate the comparison with PSV data from the global compilation^2^. The number of eliminated transitional directions is less than 5%, so we presume that the estimation of PSV is not substantially influenced by the constant VGP latitude cut-off of 45°, even if this method is not unanimously accepted^3^,^30^.

The VGPs of the selected data set have the longitudes uniformly distributed and the colatitudes exponentially distributed as required by the Fisher distribution^18^, but the distribution of directions is not Fisherian (Supplementary Fig. S6). Taking into account these results, the rest of the analysis will be done in the VGPs space. We have performed two types of reversal tests and the results showed that both tests are positive. This can be an indication for an adequate sampling of PSV. The classification of the reversal test of McFadden and McElhinny^31^ is B. The results of the bootstrap reversal test^18^ are presented in Supplementary Fig. S7.

We have computed the mean area VGP using the 76 sites selected for the analysis of PSV. The mean area VGP position and statistics are: longitude = 167.5°, latitude = 88.7°N, k = 18, A_95_ = 3.9°. This paleomagnetic pole includes both the North Geographic Pole and the European reference pole for the last 5 Ma^13^. The amount of apparent rotation and poleward displacement of the studied area, evaluated using the method of Debiche and Watson^32^, show that that no significant vertical-axis rotation and tilting are detectable after the emplacement of volcanic rocks. The result is in agreement with the post-collisional emplacement of the volcanic rocks and confirms that the studied area is suitable for the estimation of PSV. This paleomagnetic pole, which is indistinguishable from the spin axis, is in agreement with field geometry with insignificant nonzero nonaxial dipole contributions.

To describe the PSV we have used the traditional statistics^2^ of the root mean square angular deviation of VGPs (S_B_) about the geographic axis:


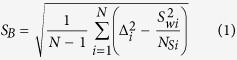


where Δ_i_ is the distance of the *i*th site’s VGP to the overall mean of the VGPs, *N* is the total number of sites, *S*_wi_ is the within-site dispersion and N_si_ is the number of samples in the ith site. The use of S_B_ is suitable for areas with the lack of detailed age control and the absence of time series of observations that are expected in working with lava flows from disparate locations^2^.

The dispersion of VGPs of volcanic rocks from the East Carpathians has been calculated for several age intervals (Supplementary Table S2). S_B_ is calculated using equation (1), and 95% confidence limits were estimated using a bootstrap resampling technique^33^. Taking into account the available number of sites and their geographical distribution (Supplementary Fig. S1), we have define several time intervals: 0.04–1.1 Ma, 1.5–2.8 Ma, 1.5–4 Ma, 4–6 Ma and 0–6 Ma. The data from the interval 0.04–1.1 Ma (27 sites) group the data obtained from the basalts of the Perşani Mountains^14^ and the youngest volcanism in the East Carpathians^34^,^35^. The data from the South Harghita Mountains (volcanic structures Luci-Lazu, Cucu and Pilişca)^13^ cover the time interval 1.5–4 Ma (48 sites). We calculated also S_B_ for a temporal subset of the South Harghita Mountains data (volcanic structures Cucu and Pilişca, Supplementary Fig. S1) covering the time interval 1.5–2.8 Ma (33 sites). The data set for 4–6 Ma is based on the data from this study supplemented with 7 sites from the norther part of the South Harghita Mountains^13^ (76 sites). To calculate S_B_ the reverse polarity data has been flipped to its equivalent normal polarity and only sites with latitude larger than 45°N or S were used for all data sets. For the 0–6 Ma data set S_B_ was computed separately for the normal and reversed polarity.

**Figure 4:**
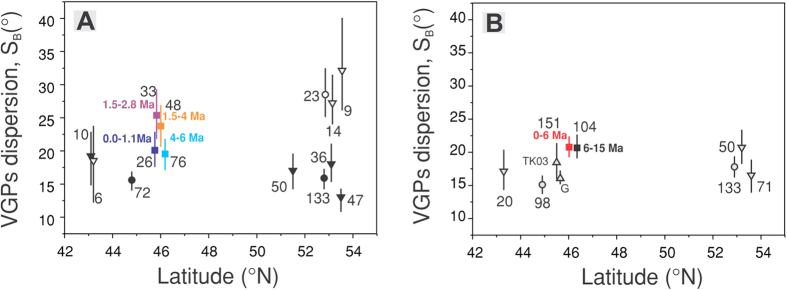
(**A**) VGPs dispersion from lavas and their 95% confidence intervals in the latitudinal band 43–54°N. Squares represent the data from the East Carpathians. Data from Johnson *et al.*^2^ are represented with full symbols for Brunhes-age normal polarity, open symbols for Matuyama-age reversed polarity: inverted triangles represent data only from TAFI studies, circles are latitudinally binned global data. Numbers near symbols represent number of sites used to calculate S_B_; (**B**) VGPs dispersion from lavas and their 95% confidence intervals for 0–6 Ma (red square, this study), 6–15 Ma^37^ (black square) and 0–5 Ma^2^ (inverted triangles represent data only from TAFI studies, circles are latitudinally binned global data) combined data sets. Numbers near symbols represent number of sites used to calculate S_B_. Triangles represent predicted dispersions^13^ for models G^38^ and TK03^37^.

Our results and the data for the Time Averaged geomagnetic Field Initiative (TAFI) studies and global compilation^2^ in the latitudinal band 42–55°N are presented in Fig. 4A. In this latitudinal band there are the nearest PSV data relevant for the sampling area latitude. The data from the TAFI studies and global compilation are presented only for the Brunhes chron and the Matuyama chron, because in this latitudinal band the data are dominated by sites which belong to these chrons.

The value of S_B_ between 4–6 Ma obtained in this study is similar to the value obtained between 0.04–1.1 Ma and is in general agreement with the data for the Time Averaged geomagnetic Field Initiative studies. However, the data set between 1.5–4 Ma have higher dispersion. The dispersion corresponding to the subset between 1.5–2.8 Ma is significant higher than other values recorded in younger or older lavas from the East Carpathians. The global compilation for the Matuyama chron^2^ shows several estimates of S_B,_ around 53°N latitude which are higher than during the Brunhes chron (Fig. 4A). The results from the East Carpathians seem to support these results showing an increase dispersion of VGPs between 1.5–2.8 Ma. The largest constraint on effective measurement of VGP dispersion is the number of available site mean data. Simulated VGP dispersion curves^36^ have shown that the number of sites means per data set required to obtain a statistical significant estimates of S_b_ must be larger than 18. Other authors^4^ consider that studies with less than 30 sites must be classified as second tier in PSV studies. As can be seen from Fig. 4A, only one Matuyama data set from the global compilation^2^ has more than 18 sites and our result from the Carpathians is based on only 33 sites. The data presented in Fig. 4a might indicate a larger dispersion of VGPs starting around 45°N during the Matuyama chron, but more data are needed at a global level in order to determine if this reflects the behaviour of the geomagnetic field, or an artefact of inadequate number of sites.

The dispersion of the combined 0–6 Ma data set (Fig. 4B) appears to show slightly higher value than most values from the global compilation^2^ in the latitudinal band 42–55°N. This global data set is mainly dominated by Brunhes age sites, but our data set has a better coverage of the whole time interval. We presume that the observed differences probably reflect this aspect. S_B_ between 0 and 6 Ma is statistical identical with S_B_ between 6 and 15 Ma obtained from volcanic rocks of the Columbia River Basalt Group (USA)^37^. Both S_B_ values are closer to the predicted dispersion from statistical geomagnetic field model TK03^38^ than that from model G^39^. Significant difference between the reversed and normal polarity dispersion parameters is reported for the age interval 6 to 15 Ma^36^. Such differences are not observed in our data set between 0 and 6 Ma, because the data sets with normal and reversed polarity have identical Sb values (Supplementary Table S2). The data presented in Fig. 4B suggest that the geomagnetic field behaviour averaged over 6 Ma or more is constant at least around 46°N.

## Conclusions

In this study, we have measured paleomagnetic directions recorded by lava flows erupted between 4–6 Ma in the East Carpathians in order to augment and improve the observations relevant to understanding the time averaged character of the Earth’s magnetic field. Eighty lava flows gave stable paleomagnetic directions and passed the statistical criteria for PSV studies. After omitting sites with low latitude poles (<45°), the remaining 76 sites have normal and reversed mean directions that are antipodal at the 95% confidence level. The distribution of the VGPs is Fisherian and the mean pole position is indistinguishable from the spin axis. The secular variation described by the VGP angular standard deviation for these sites is 18.8°. This value is in general agreement with the data for the Time Averaged geomagnetic Field Initiative studies in the same latitudinal band. The new data set was analysed together with previous results from the same area obtained from 0.04–4 Ma old lava flows. This analysis has shown that the dispersion recorded by 1.5–2.8 Ma lava flows is significant higher than other values recorded in younger or older lavas from the East Carpathians. High values of dispersion during the Matuyama chron are also reported around 50°N in the global paleosecular variation data set, but more data are needed at a global level to determine if this reflects the behaviour of the geomagnetic field, or an artefact of inadequate number of sites. For the combined 0–6 Ma data set, the dispersion appears to show slightly higher value than most values from the global compilation in the latitudinal band 42–55°N. This value is closer to the predicted dispersion from statistical geomagnetic field model TK03^38^ than that from model G^39^. The present study of the East Carpathians volcanic rocks brings new data from the southeastern Europe which can be considered in the global databases for time averaged field and paleosecular variation from lavas in the last 6 Ma.

## Methods

Laboratory analyses were carried out in the Paleomagnetic Laboratory at the University of Bucharest. Standard palaeomagnetic specimens (11 cm^3^) were cut from each core. Remanent magnetizations were measured using a JR6A spinner magnetometer (AGICO). AF demagnetization was done using a Magnon International static AF demagnetizer. AF demagnetization was performed in steps from 0 to 200 mT, with 8–15 steps per specimen. Thermal demagnetization was performed with a home build heater (triple mumetal shields, non-inductive processor control furnace, residual magnetic field less than 5 nT). The heater and the magnetometer are installed inside a set of three Helmholts coils used to reduce geomagnetic field in the working area to less than 300 nT. The susceptibility variation upon thermal treatment was measured on Bartigton MS2B system. Thermal demagnetization was performed in 8–12 steps between 150 and the maximum unblocking temperature. Statistical analysis of directional data was done using Lisa Tauxe’s PmagPy-3.24 software package^32^. The hysteresis properties of at least one specimen per site were measured at room temperature using a VSM model 3900 (Princeton Measurements) with a maximum applied field of 1 T. The saturation magnetization (*M*s), saturation remanent magnetization (*M*rs) and coercive force (*B*c) values were calculated after correction for the paramagnetic contribution. The coercivity of remanence (*B*cr) and the ratio between isothermal remanent magnetization at 300 mT and *M*rs (S ratio) were determined by applying a progressively increasing backfield after saturation. The FORC measurements were made using the irregular FORC protocol^40^. The field dependence of the magnetic susceptibility at 50 and 700 Am^–1^ was determined for a specimen per site using the MFK1A kappabridge (AGICO). For representative specimens the temperature dependence of magnetic susceptibility was measured with a CSL apparatus from liquid nitrogen temperature to room temperature and with CS3 apparatus from room temperature to 700 °C. Both instruments were coupled with the MFK1A kappabridge. The heating–cooling cycle above room temperature was performed in argon atmosphere.

## Additional Information

**How to cite this article**: Vişan, M. *et al.* Palaeomagnetism of the Upper Miocene- Lower Pliocene lavas from the East Carpathians: contribution to the paleosecular variation of geomagnetic field. *Sci. Rep.* **6**, 23411; doi: 10.1038/srep23411 (2016).

## Supplementary Material

Supplementary Information

## Figures and Tables

**Figure 1:**
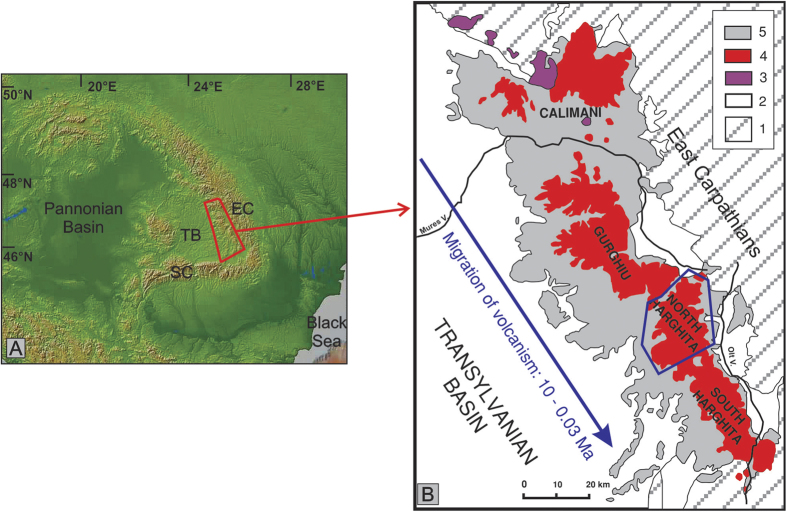
(**A**) Location of the Călimani-Gurghiu-Harghita volcanic chain in the Carpatho -Pannonian area (SC = South Carpathians; EC = East Carpathians; TB = Transylvanian Basin). Map was created using GMRT Map tool (www.marine-geo.org/portals/gmrt/)^41^; (**B**) Location of the sampling area (blue contour) in the schematic map of the Calimani – Gurghiu –Harghita volcanic chain. Symbols: 1. East Carpathian nappes system; 2. Sedimentary basins; 3. Pre-volcanic intrusions; 4. Lava flows; 5. Volcaniclastic facies. Map was created with QGIS 2.8 open-source software (http://www.qgis.org/) using the limits of geological formations digitized from the Geological Map of Romania – scale 1:200000^42^,^43^,^44^.
