# Supplementary material for: Palaeomagnetism of the Upper Miocene- Lower Pliocene lavas from the East Carpathians: contribution to the paleosecular variation of geomagnetic field
Source: Sci Rep. 2016 Mar 21;6:23411. doi: 10.1038/srep23411 (PMC4800496; doi:10.1038/srep23411)
Supplement: Supplementary Information [file srep23411-s1.pdf]

Paleomagnetism of the Upper Miocene- Lower Pliocene lavas from the East Carpathians: contribution to the paleosecular variation of geomagnetic field

Mădălina Vişan[1], Cristian G. Panaiotu[2*], Cristian Necula[2], Anca Dumitru[2]

[1] Institute of Geodynamics, Romanian Academy, Jean-Luis Calderon 19-21, 020032, Bucharest, Romania.

[2] Paleomagnetic Laboratory, Faculty of Physics, University of Bucharest, Atomiştilor 405, Măgurele, Ilfov, Romania.

*Corresponding author, Cristian G. Panaiotu: cristian.panaiotu@g.unibuc.ro

Supplementary figures

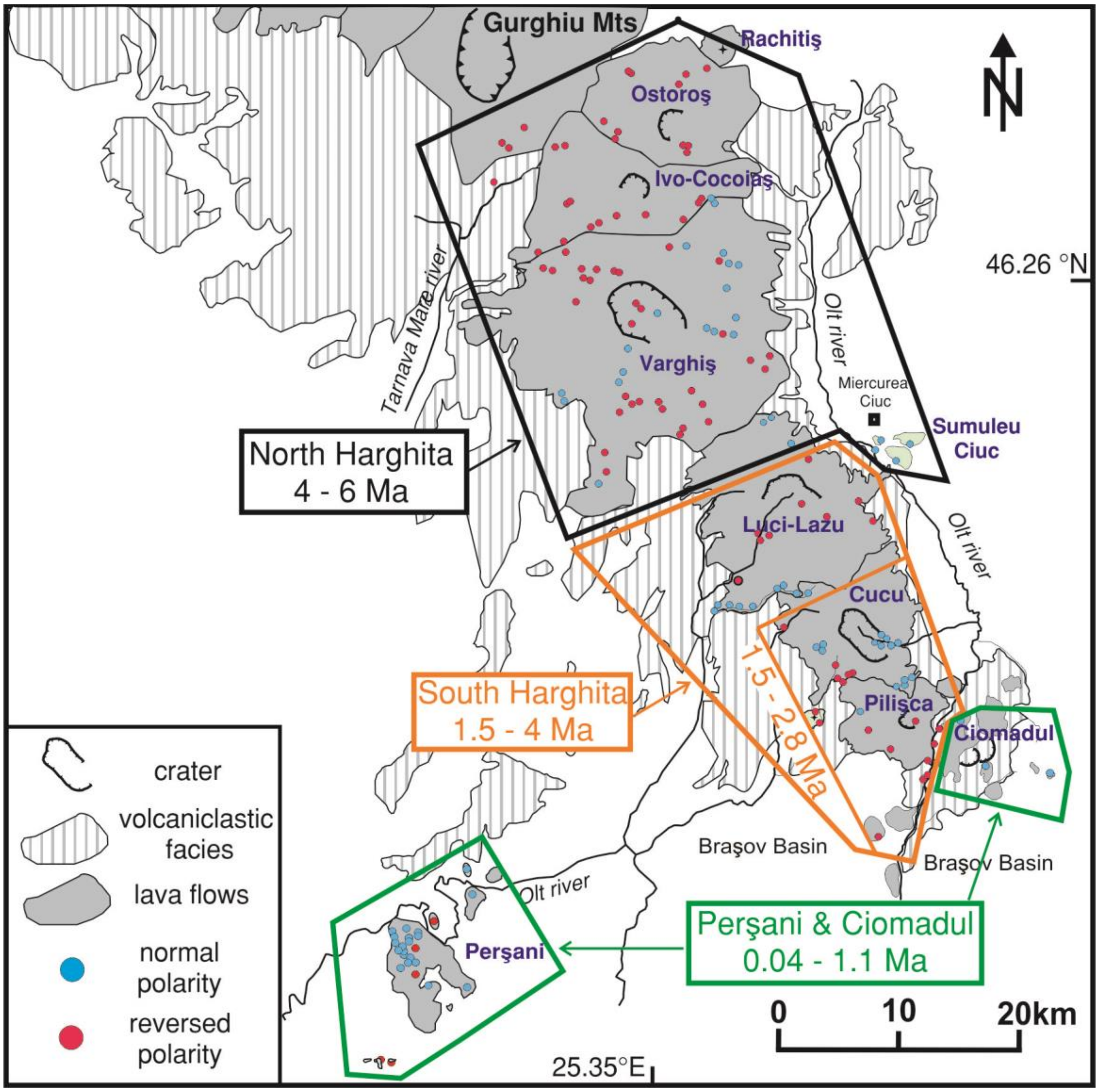

Figure S1 Location of paleomagnetic sites and their magnetic polarity. Names of the main volcanic structures[11] are written in bold blue. Sites from the North Harghita are from this study with exception of 4 sites from the Sumuleu-Ciuc volcanic structure and 3 sites from the northern part of the Luci-Lazu volcanic structure which are from previous study[13]. Sites from the South Harghita, Ciomadul and Perşani are from previous studies[13, 14]. Map was created using QGIS 2.8 open-source software (http://www.qgis.org/). Limits of geological formations are from the Geological Map of Romania – scale 1:200000[41].

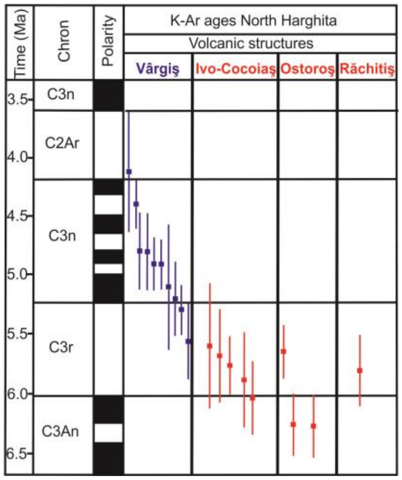

Figure S2 K-Ar ages[16] of volcanic structures from the North Harghita Mountains. Răchitiş, Ostoroş and Ivo-Cocoiaş volcanic structures are in the northern part of the area and the Virghiş volcanic structure is in the south (see Supplementary Fig. S1).

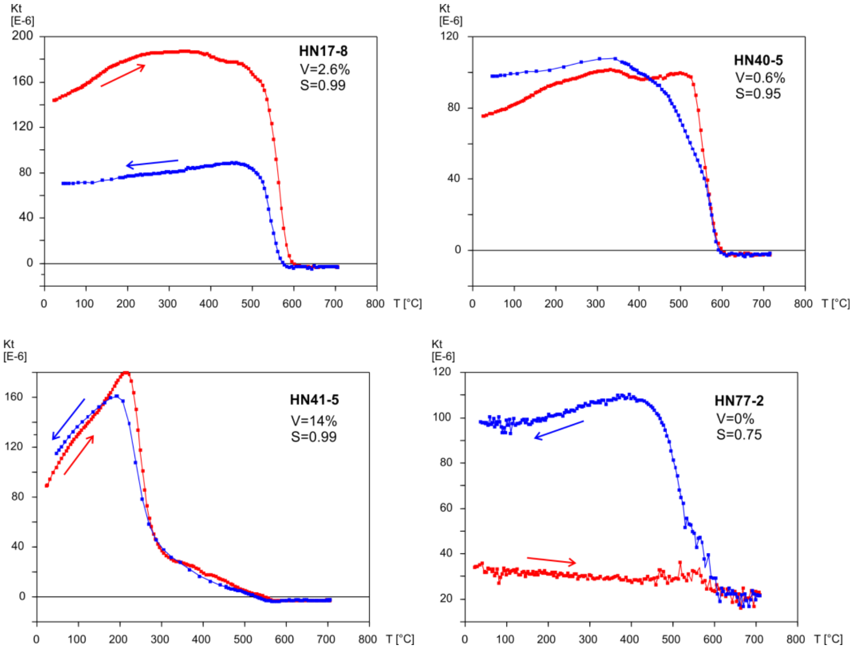

Figure S3 Examples of magnetic susceptibility (Kt) variation with temperature (T). Heating and cooling curves are plotted in red and blue, respectively. For each samples we report the S ratio and the V parameter.

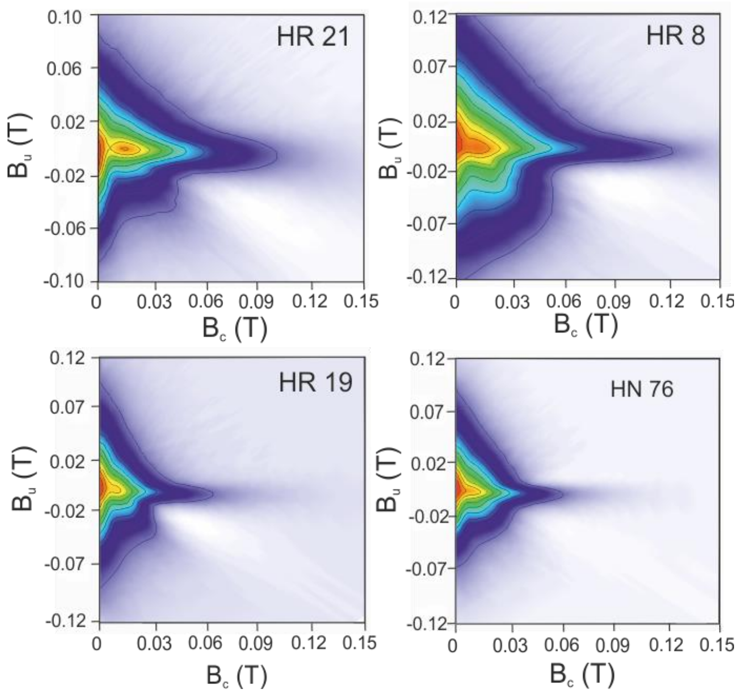

Figure S4 FORC diagrams for the samples marked with yellow circles in Fig. 2b. The FORC measurements were made using the irregular FORC protocol[39]. To generate the irregular grid we measured hysteresis loops for each samples with 4 mT increment and a saturating field of 500mT. The hysteresis loops where corrected for drift and para/diamagnetic contribution using the irregularFORC package[39]. All the hysteresis loops are closed at about 400 mT and this field was taken as saturating field for the irregular FORC measurements. We measured 80 FORCs for each sample and the averaging time was set to 200 ms. The HN21 sample was smoothed using average filter[39] with SF=2, while all the other three samples were smoothed using the same average filter and SF=1.

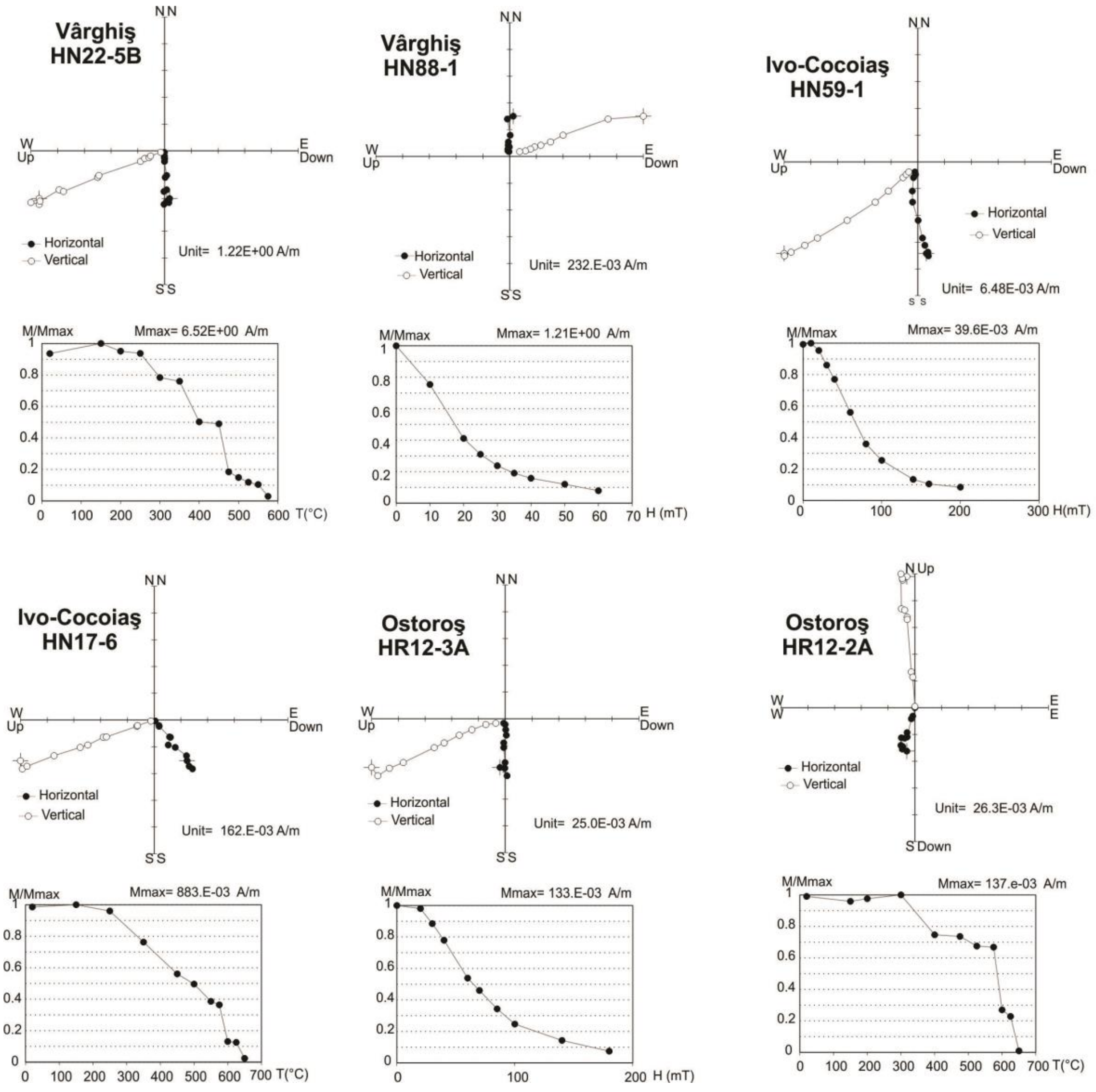

Figure S5 Examples of evolution of direction and intensity of the natural remanent magnetization during AF and thermal demagnetization.

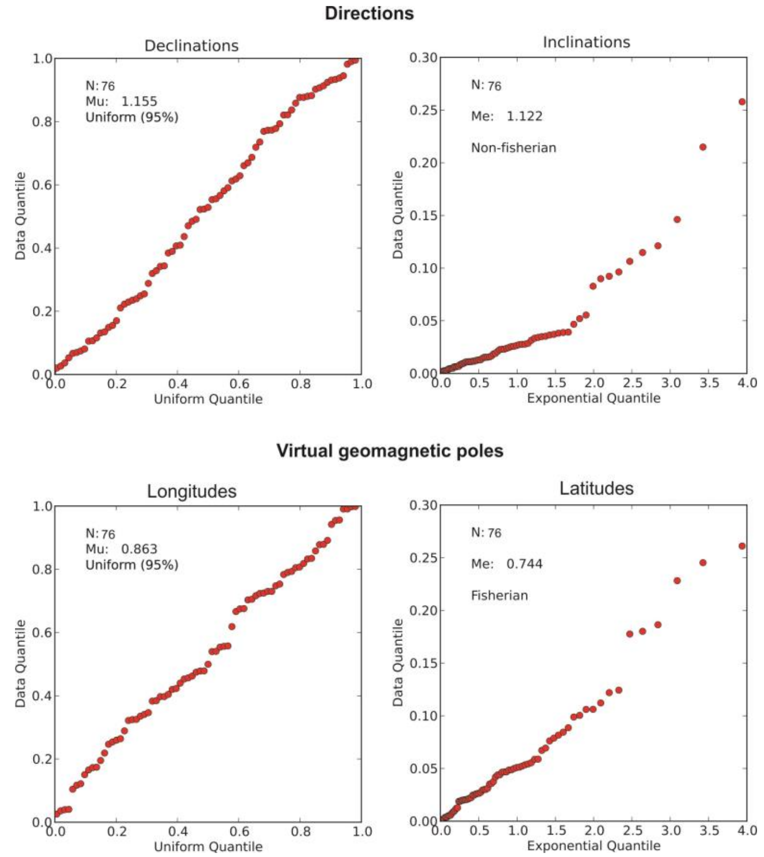

Figure S6 Q – Q plots and results of statistical tests for the distribution of directions and virtual geomagnetic poles (only sites with VGP latitude > 45°N or 45°S). Analyses and plots were done using using the PmagPy-3.24 software package[32].

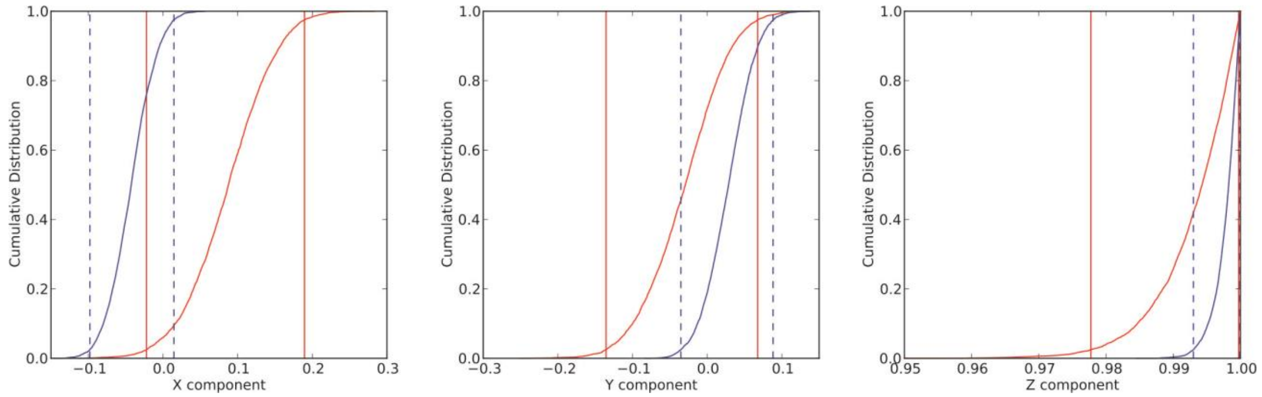

Figure S7 Bootstrap reversal test diagrams[18] of VGPs (only sites with VGP latitude > 45°N or 45°S). The means of the normal and reversed modes cannot be distinguished at the 95% level of confidence, indicating a positive bootstrap reversal test. Analyses and plots were done using the PmagPy-3.24 software package[32].

Supplementary Tables

Table S1 Paleomagnetic results of lava flows emplaced between 4 and 6 Ma

Site	Site GPS coordinates			Site mean direction and statistics					VGP	
	Latitude (°N)	Longitude (°E)	Altitude (m)	Dec (°)	Inc (°)	N	k	α_{95} (°)	Plong (°)	Plat (°)
Southern tip of the Gurghiu Mountains										
G17	46.539717	25.4313	1072	163.5	-71.4	6	123	6	164.4	-76.0
G18	46.536367	25.43695	946	179.4	-75.6	7	216	4	204.5	-73.8
G19	46.512367	25.423467	851	149.9	-72.7	6	187	5	158.9	-68.5
G24	46.549433	25.452883	946	160	-76.8	5	86	8	181.7	-68.7
Ostoroş volcanic structure (North Harghita Mountains)										
HR4	46.532367	25.62595	1092	202.7	-41.9	6	75	8	338.7	-61.2
HR5	46.5853	25.644717	1000	188.7	-57.5	6	281	4	6.7	-81.1
HR6	46.582633	25.625583	1045	171.3	-51.1	6	559	3	66	-71.8
HR7	46.575833	25.618533	1190	180.1	-45.3	6	932	2	38.4	-69.8
HR8	46.584483	25.571033	975	194.8	-49.5	6	1008	2	357	-72.1
HR9	46.583367	25.573467	1010	200.7	-49.6	6	379	3	343.2	-69.8
HR11	46.540283	25.558333	1171	210.3	-57.7	5	157	6	310.3	-69.7
HR12	46.544683	25.561267	1120	184.9	-73.2	6	160	5	205.3	-77.7
HR13	46.55155	25.548617	1014	208.6	-49.7	5	65	10	321.3	-62.7
HR21	46.5365	25.623833	1072	171.5	-58.3	6	265	4	83.1	-77.6
HR22	46.5363	25.62705	1047	205.7	-61.5	6	424	3	303	-74.8
Ivo-Cocoiaş volcanic structure (North Harghita Mountains)										
HR15	46.498517	25.515167	953	184.7	-64.1	6	136	6	43.6	-89.3
HR17	46.499567	25.518067	981	195.2	-64.7	6	61	9	290.6	-83
HR18	46.536183	25.510417	896	148.5	-67.4	7	304	4	137.8	-65.7
HR19	46.535733	25.500967	844	286.9	-82.4	6	96	7	225.3	-41.6
HN 57	46.472817	25.510367	944	204.3	-41.3	7	176	5	342.9	-62.0
HN59	46.489417	25.560833	1224	169.3	-57.4	5	57	10	72.2	-78.4
HN 60	46.4848	25.544017	1161	178.9	-20.8	6	97	7	35.8	-53.9
HN 61	46.482583	25.5363	1076	132.9	-58.6	5	116	7	124	-51.8
HN 63	46.4659	25.485867	826	210.7	-68.9	6	224	5	266.8	-71.8
HN 74	46.50135	25.640617	927	213.9	-73.8	6	265	4	246.8	-68.4
HN 75	46.499333	25.6387	963	194.2	-75.5	6	125	6	226.9	-72.0
HN 76	46.487383	25.623733	1058	198.6	-68.8	5	70	9	263.6	-76.7
HN 77	46.490183	25.587933	1248	198.2	-64.4	5	57	10	290	-77.5
Vârghiş volcanic structure (North Harghita Mountains)										
HN17	46.367033	25.640933	1182	196.6	-64.3	5	69	9	297	-85.4
HN18	46.376233	25.630933	1278	177.2	-54.5	5	423	4	37.1	-78.4
HN22	46.390317	25.700267	913	188.8	-66.3	5	511	3	218.9	-87.6

HN23	46.395517	25.686667	959	210.4	-44.6	5	31	14	339.8	-64.1
HN24	46.39817	25.705233	916	197.4	-49.6	8	13	16	323	-63.4
HN26	46.367117	25.576083	1036	169.6	-62.5	7	108	6	112.8	-75.4
HN27	46.36475	25.603167	1319	164.1	-40.4	5	50	11	59.8	-63.4
HN28	46.367717	25.599383	1311	255.9	-49.6	6	130	6	282.3	-30.8
HN29	46.368883	25.581283	1078	185.5	-67.1	6	74	8	180.4	-86.2
HN30	46.3725	25.56945	1003	180.9	-55.5	7	65	8	55.3	-78.4
HN31	46.3742	25.584767	953	190.5	-60.3	6	160	5	38.4	-84.8
HN32	46.432283	25.57945	1500	202.1	-63.2	6	50	10	293.8	-74.5
HN34	46.429733	25.58365	1449	173.6	-56.1	6	112	6	9	-79.8
HN35	46.41923	25.57483	1244	174.1	-68	6	117	6	167.7	-83.9
HN36	46.33525	25.5488	847	185.2	-54	6	123	7	5.2	-77.5
HN37	46.38165	25.56307	1028	357	81.8	6	239	4	21.4	62.3
HN38	46.38823	25.56667	1054	314.7	84.2	5	38	13	11.1	53.0
HN39	46.40362	25.57168	1154	309.5	56.1	6	107	7	302	48.9
HN40	46.31442	25.54247	761	340.8	75.5	6	122	6	352	67.6
HN41	46.32293	25549633	795	194.7	-70.3	7	68	8	249.4	-77.7
HN42	46.35185	25.643383	1130	186.8	-31.4	6	761	2	12.5	-60.1
HN43	46.34813	25.62037	1056	160.2	-62.2	7	325	3	114.6	-73.0
HN44	46.35127	25.6227	1106	162.4	-59	7	207	4	100.7	-73.0
HN47	46.4268	25.59837	1358	359.1	63.4	5	131	7	228.9	88.4
HN51	46.4522	25.56365	1459	182.3	-64.9	6	260	4	129.2	-88.1
HN52	46.45328	25.5588	1465	207.9	-53.1	6	209	5	316.8	-65.3
HN53	46.45502	25.54128	1183	214.5	-67.7	5	253	6	272.5	-69.5
HN54	46.45502	25.52802	1180	209.6	-70.9	6	673	3	257.3	-71.7
HN58	46.47312	25.55062	1225	186	-52.7	5	609	3	4.4	-76.1
HN62	46.46598	25.51202	939	168.9	-58.2	6	59	9	86.4	-76.6
HN64	46.45617	25.49037	872	204.6	-72.8	6	227	5	244.5	-72.9
HN65	46.45422	25.49947	957	173.6	-70.2	6	190	5	168.7	-79.8
HN66	46.43393	25.5221	1093	149.6	-68.8	5	132	7	142.6	-67.1
HN67	46.4492	25.52938	1142	194.2	-66.1	5	250	5	278.2	-80.2
HN68	46.44778	25.53587	1199	173.5	-52.1	5	47	11	47.6	-75.4
HN69	46.36183	25.51108	866	25.9	61.7	5	441	4	117	71.3
HN70	46.36368	25.50965	863	19.8	69.6	5	483	4	79.2	75.5
HN71	46.30045	25.58963	760	234.7	-54.6	6	410	3	290.8	-47.9
HN79	46.46893	25.62638	1193	8	55.1	6	113	6	173.7	77.6
HN86	46.45987	25.65713	1115	189.2	-50.8	6	355	4	357.1	-73.5
HN88	46.4586	25.66688	1058	2.5	67.1	6	283	4	0.6	86.2
HN89	46.45723	25.67517	1038	9.3	66.3	6	183	5	92.3	83.3
HN90	46.4427	25.6648	1108	331.6	70.6	5	110	7	331.3	70.3
HN91	46.4198	25.66727	1128	24.8	68.5	5	575	3	85.8	76.0

HN92	46.41332	25.65833	1096	202.6	-58.8	5	546	3	317.6	-75.4
HN93	46.41432	25.6524	1188	17.6	25.5	5	1653	2	175.7	53.8
HN94	46.41675	25.64515	1218	355.5	57.9	5	100	8	230	81.5
HN96	46.41243	25.67092	1073	22.7	62.1	5	86	8	122.8	77.1
HN 72	46.50382	25.65048	889	354.7	66.5	6	213	5	333.1	85.6
HN 73	46.50477	25.6497	929	351.9	70.2	4	585	4	355	80.6
HN19*	46.3604	25.69692	927	345.6	67.7	6	227	5	325	79.6
HN56*	46.3641	25.70053	900	359.7	68.9	5	1031	2	24.1	84
HN81*	46.34593	25.7158	950	335	61.9	5	616	3	294.5	72
Sumuleu – Ciuc volcanic structure										
HN46*	46.34587	25.809	707	307	38	7	195	4	284.1	40.6
HN45*	46.33805	25.80258	659	337	60.5	7	666	2	322.5	72.8
HN25*	46.34067	25.84	762	287.4	30	7	83	7	294.8	23.5
H10*	46.32874	25.82281	685	325.4	44.7	6	260	4	271.7	56.1

* Sites from previous study[13].

Dec and Inc: declination and inclination of site mean; N: number of samples.

k and α_{95}: precision parameter and semi-angle of 95 per cent confidence (Fisher statistics[26]).

Plong and Plat: longitude and latitude of VGP.

Table S2 VGP dispersions of lava flows from the East Carpathians

Latitude (°N)	Age (Ma)	Polarity	N	S_B (°)	S_B^{lo} (°)	S_B^{hi} (°)
46.02 - 46.36	0.04 - 1.1	All combined	27	20.3	16.8	23.5
45.89 – 46.21	1.5 – 2.8	All combined	32	25.2	21	29.2
45.89 - 46.33	1.5 - 4	All combined	48	23.5	20.5	26.5
46.35 – 46.58	4 - 6	All combined	76	18.8	16.5	21.1
46.02 – 46.58	0 - 6	All combined	151	20.5	18.9	22.1
46.02 – 46.58	0 - 6	Normal	66	20.6	18.0	22.9
46.02 – 46.58	0 - 6	Reversed	85	20.6	18.3	22.7

N: number of sites. S_B: the between-site VGP dispersion, along with 95% confidence limits (S_B^{lo}, S_B^{hi})
